# Molecular diagnosis and genotyping of *Pneumocystis jirovecii* in bronchoalveolar lavage samples obtained from patients with pulmonary disorder

**DOI:** 10.18502/cmm.5.3.1741

**Published:** 2019-09

**Authors:** Abdolmajid Fata, Bahareh Abdollahi, Fariba Rezaeetalab, Davood Attaran, Mohsen Najjari, Mohammad J. Najafzadeh

**Affiliations:** 1 Department of Parasitology and Mycology, Faculty of Medicine, Mashhad University of Medical Sciences, Mashhad, Iran; 2 Cutaneous Leishmaniasis Research Center, Mashhad University of Medical Sciences, Mashhad, Iran; 3 Department of Internal Medicine, Faculty of Medicine, Mashhad University of Medical Sciences, Mashhad, Iran; 4 Lung Diseases Research Center, Mashhad University of Medical Sciences, Mashhad, Iran; 5 Cancer Molecular Pathology Research Center, Mashhad University of Medical Sciences, Mashhad, Iran

**Keywords:** Bronchoalveolar Lavage (BAL), Iran, Nested-PCR, Pneumocystis jirovecii, Pneumocystis pneumonia (PCP)

## Abstract

**Background and Purpose:**

Pneumocystis pneumonia (PCP) is one of the most common and life-threatening fungal diseases in patients with human immunodeficiency, treated with immunosuppressive medications. Immunocompetent people can also be a spreading agent for PCP. Regarding this, the aim of the present study was to diagnose and identify *Pneumocystis jirovecii* in bronchoalveolar lavage (BAL) samples obtained from patients with pulmonary disorder using a molecular method.

**Materials and Methods:**

For the purpose of the study, BAL samples (n=138) were collected from patients, undergoing bronchoscopy at the different departments of university hospitals affiliated to Mashhad University of Medical Sciences, Mashhad, Iran, during a period of one year (i.e., April 2014 until May 2015). Giemsa staining and molecular identification were carried out for each sample. The samples were also subjected to nested polymerase chain reaction (PCR), sequencing, and genotyping based on mitochondrial ribosomal large subunit (*mtLSU* rRNA) of *P. jirovecii*. The phylogenic tree was constructed by MEGA6 software.

**Results:**

The results of direct microscopic examination revealed the presence of *P. jirovecii *in 3 (2.2%) out of 138 samples; in addition, nested PCR and sequencing led to the detection of species in 17 (12.3%) samples. Out of patients with positive results, 10 (25%) and 7 (7.1%) cases were immunosuppressed and immunocompetent, respectively. The most common clinical symptoms among patients with pneumocystis were fever, dyspnea, and dry cough. In addition, genotypes III and II were the dominant genotypes in our dataset.

**Conclusion:**

Nested PCR and sequencing methods showed higher sensitivity and specificity as compared with a direct staining technique. Genotype III was identified as the most dominant type in patients with pulmonary disorder in Mashhad.

## Introduction

With the rising spectrum of infectious organisms, the incidence of pulmonary disorders has also been increased [1]. *Pneumocystis jirovecii, *formerly known as *Pneumocystis carinii*, can be found in air dust, and therefore transmitted through inhalation [2, 3]. Serological evidence could be positive for many people during their childhood [4]. *Pneumocystis jirovecii *is one of the most common causes of respiratory infections in people infected with human immunodeficiency virus (HIV) and could lead to pneumocystis pneumonia (PCP) [5, 6]. 

The epidemiological aspects of pneumocystis infections in HIV-positive people have been changed after the introduction and widespread use of highly active antiretroviral therapy (HAART). In addition to HAART, the use of anti-pneumocystis prophylaxis has resulted in a decrease in the morbidity and mortality of PCP. In the same vein, the recent retroviral treatments and prophylaxis against HIV have led to the reduction of the morbidity and mortality of HIV patients, infected with PCP [7]. 

Pneumocystis pneumonia can occur in patients with other immunodeficiencies, such as those with blood malignancies or recipients of immunosuppressive medications [8]. This infection can also affect healthy individuals [9] and patients with different pulmonary diseases [10, 11]. Colonization is an important step in the transmission of *P. jirovecii* [12], a few numbers of which can pave the way for pulmonary inflammation and affect the prevalence of this pulmonary disease [13]. 

To diagnose such kind of pulmonary diseases, especially in immunodeficient people, it is crucial to set a new, fast, and reliable method [14]. Bronchoalveolar lavage (BAL) is the best sample for the diagnosis of PCP [12]. There have been different studies on PCP and its causative agent worldwide; however, the numbers of such studies have been limited in Iran [11, 15-18]. Regarding this, the purpose of the present study was to isolate and identify *P. jirovecii* in BAL samples obtained from patients with pulmonary disorder admitted to the University Hospitals of Mashhad. 

## Materials and Methods

***Patients and sampling***

During April 2014 to May 2015, a cross-sectional descriptive study was performed on 138 BAL samples obtained from patients with pulmonary disorders, admitted to the university hospitals of Mashhad University of Medical Sciences (i.e., Imam Reza and Ghaem hospitals), Mashhad, Iran. This study was approved by the Ethics Committee of Mashhad University of Medical Sciences, Mashhad, Iran (Ethical code: IR.MUMS.REC.1394.31). 

The patients who had taken antibiotics (e.g., trimethoprim/sulfamethoxazole, pentamidine, atovaquone, dapsone, pyrimethamine, and trimetrexate) during the last 2 months were excluded from this study. A questionnaire containing demographic and clinical information was completed for each patient. According to these data, the patients were divided into two groups of immunodeficient and immunocompetent. In this regard, Elderly solid-organ receivers, HIV-positive individuals, patients with malignancy, and corticosteroids users were grouped as immunodeficient. 

For each patient, 10-12 ml BAL was obtained by a physician in a sterile test tube and sent to the Mycology Lab of Imam Reza Hospital. Direct smears were prepared from the sediment of BAL samples and stained by Giemsa stain. The remaining materials were kept at -20°C to be further used in molecular tests. Nested polymerase chain reaction (PCR), sequencing, and genotyping were performed on the mitochondrial ribosomal large subunit (*mtLSU*) rRNA gene of *P. jirovecii.*

***DNA extraction and nested polymerase chain reaction***

The DNA extraction was accomplished by means of the Tissue PrimePrep Genomic kit (Gente Bio brand, Korea). The PCR was performed using *mtLSU *rRNA gene. The primers used in the first step included PaZ102-H and Paz102-E. Furthermore, the primers of the second step entailed PaZ102-Y and PaZ102-X [3]. The PCR reaction tubes contained 10 mM Tris-HCl (pH=8.0), 50 mM KCl, 1.5 mM MgCl_2_, 0.2 mM of each dNTP, 300 ng of each primer, 1.5 U Platinum Taq Polymerase, and 25--50 ng of genomic DNA in a volume of 50 μL. 

The reaction cycles included an initial cycle of 5 min at 96°C, followed by 40 cycles of 96°C for 30 sec, 60°C for 35 sec, and 72°C for 40 sec. The PCR products were analyzed using 2% agarose gel electrophoresis and observed under ultraviolet transilluminator. These products were directly sequenced with ABI 310 Genetic Analyzer Applied Biosystems, using the ABI Prism BigDye Terminator Cycle Sequencing Ready Reaction Kit (Applied Biosystems; version 1.1). The data were analyzed in SeqMAN software (DNASTAR, Wisconsin, USA). Phylogenetic trees were constructed and edited using the maximum likelihood implemented in MEGA software (version 6) with 1,000 replicates [19]. Alignments were available upon request. 

## Results

In this study, 138 patients, including 67 males (48.6%) and 71 (51.4%) females within the age range of 16-95 years (57.06±16.58 years) were examined by bronchoscopy. A total of 109 (79%) patients referring to bronchoscopy department were hospitalized, and 29 (21%) subjects were not hospitalized (Table 1).* Pneumocystis jirovecii *was diagnosed in 3 (2.2%)  

**Table 1:** Frequency distribution of pulmonary disorders in patients admitted to the different wards of Imam Reza and Ghaem hospitals, Mashhad, Iran

**Hospital ward**	**All patients**		**Patients with ** ***Pneumocystis jirovecii***	
	**No.**	**%**	**No.**	**%**
Pulmonary diseases	31	28.4	3	20
Intensive care unit	23	21.1	3	20
General	13	11.9	2	13.3
Infectious diseases	10	9.2	3	20
Emergency	8	7.3	1	6.6
Nephrology	8	7.3	1	6.6
Rheumatology	6	5.5	1	6.6
Gastrointestinal	4	3.7	0	0
Hematology	2	1.8	0	0
others	4	3.7	1	6.6
Total	109	100	15	100

samples by Giemsa staining, while nested PCR was positive for 17 (12.3%) samples, including the 3 mentioned samples (Figure 1). Sequences were deposited in the GenBank under the accession numbers of MH645495-MH645511.

As indicated in the phylogeny tree displayed in Figure 2, *Pneumocystis carinii* species *muris* U20169 was chosen as the outgroup taxa. Table 2 presents the clinical signs of all patients, as well as those of *P. jirovecii -*infected patients. Genotypes III, II, and I were detected in the studied population (100%; i.e., 17 out of 17 PCR-positive BAL samples). Genotype III was identified as the dominant genotype (n=10), followed by genotypes II (n=6) and I (n=1; Figure 3). 

**Figure 1:**
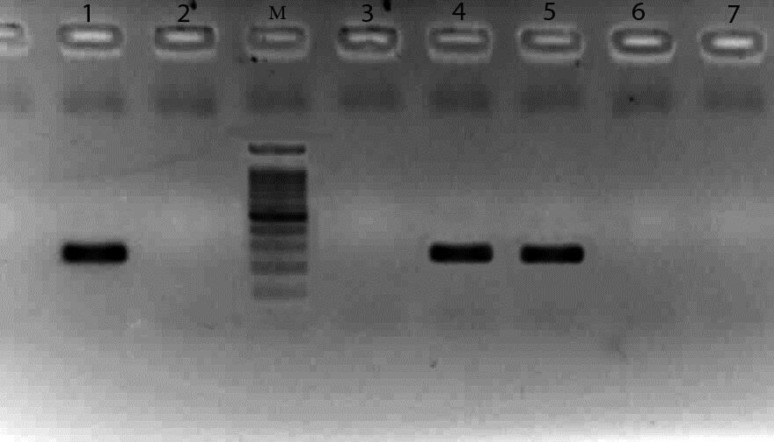
Electrophoresis results of polymerase chain reaction products in agarose gel 1.2% (Fermentas marker 100 bp) (No 1 and 2 are positive and negative controls, respectively, M: Marker, the 260 bp band of the *mtLSU* rRNA gene was considered positive for *P. jirovecii.* (No 4 and 5) and no 3, 6, and 7 are negative cases.)

**Figure 2:**
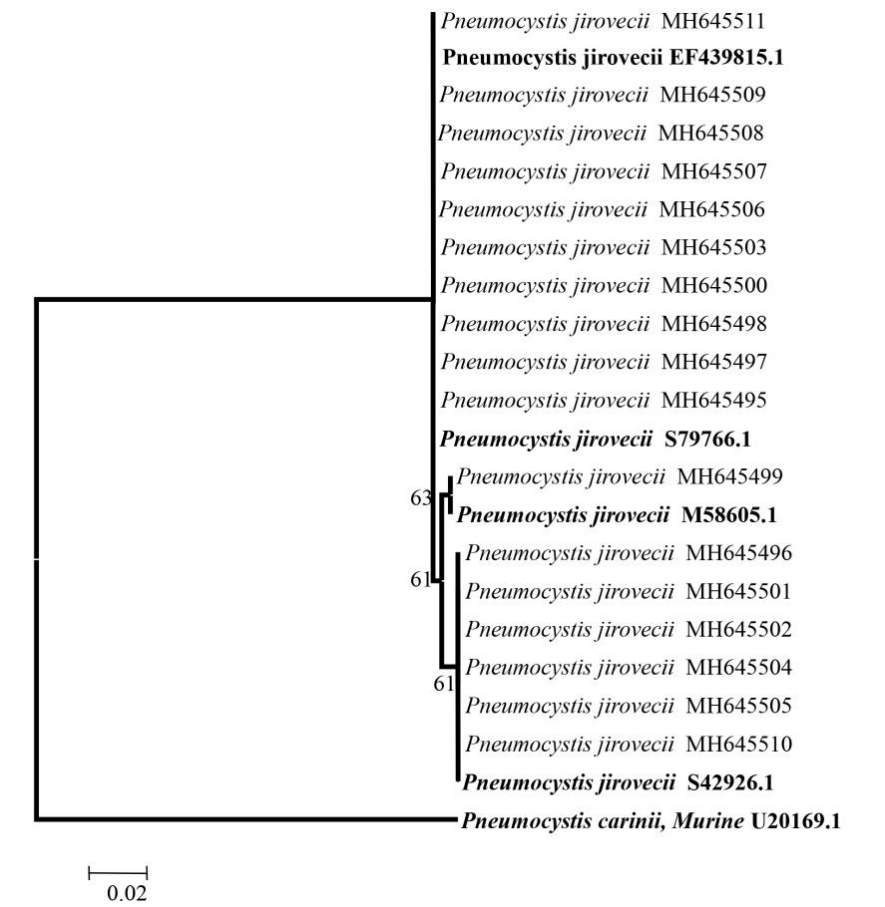
Maximum likelihood tree based on LSU sequence of *Pneumocystis jirovecii *(*Pneumocystis carinii U20169.1* was used as outgroup. Boot strap values above 50% are shown on the branches. Genbank accession numbers are in front of the isolates. Bold isolates were selected from Genbank.)

**Table 2:** Distribution frequency of clinical signs in pulmonary patients and patients with *Pneumocystis* *jirovecii *in Imam Reza and Ghaem hospitals in 2014-2015

**Clinical signs**	**All Patients (n=138)**		***P. jrovecii*** ** patients (n=17)**	
	**Frequency**	**Percentage**	**Frequency**	**Percentage**
Sputum cough	74	53.6	5	29.4
Dry cough	25	18.1	7	41.2
Dyspnea	98	71.1	9	52.9
Fever	66	47.8	10	58.8
Loss of weight	43	31.2	4	23.5
Night sweat	25	18.1	5	29.4

**Figure 3:**
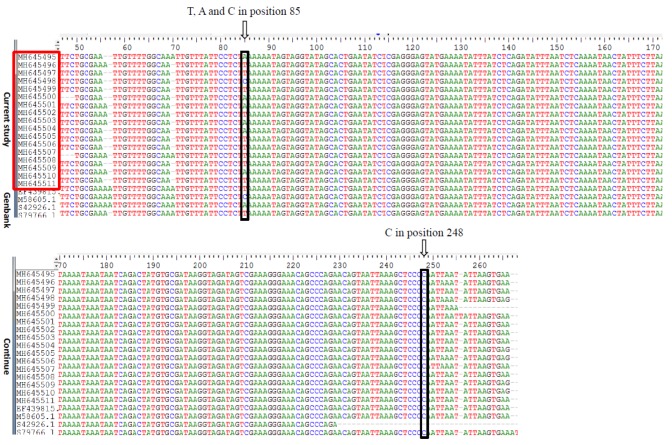
Aligned Genbank accession number of *Pneumocystis* *jirovecii mtLUS* rRNA gene from the current study and Genbank (They have nucleotide variation at only position 85, confirming genotypes III, II, and I. They have a ‘C’ at position 248 and a ‘T’ at position 289 [not shown].)

There was no relationship between age, gender, hospitalization or non-hospitalization and infection. In addition, cough, dyspnea, and fever were identified as common clinical signs. Seven patients (41.1%) out of 17 had a dry cough, 5 (6.3%) cases of whom were non-Iranian but permanent residents of Mashhad. Out of these 5 subjects, 2 (40%) cases had pneumocystis. Furthermore, 10 (25%) out of 40 patients with immunodeficiency or reduced immunity and 7 (7.1%) out of 98 patients without immunodeficiency had pneumocystis. 

In our study,* P. jirovecii *was seen in 3 (43%) transplanted patients, 1 (5.2%) HIV-positive patient, 1 (5.2%) subject with malignancy, and 5 (38%) immunodeficient cases on corticosteroid therapy. Among 138 patients with pulmonary disease, 32 (23.2%) cases had diabetes, and pneumocystis was observed in 6 (18.8%) patients. In addition, out of 51 (38.8%) subjects that used cigarette and drugs (addictive substances), 5 cases had pneumocystis. 

## Discussion

The standard method for the detection of PCP is the microscopic observation of pulmonary samples. To date, different staining methods, such as Giemsa, Gomori Methenamine-Silver (GMS), and Methylene Blue, have been used to this end [6]. Traditional methods rely on the technician's experience and morphological recognition of this fungus; therefore, in most cases, the diagnosis is not successful. Nonetheless, the conventional methods are considered the gold standard for diagnosis. 

On the other hand, molecular methods, like nested PCR and sequencing, require advanced laboratory equipment and expert personnel [20]. Nested PCR and sequencing have higher efficiency in recognizing the presence of *P. jirovecii *DNA in immunodeficient patients than in healthy people [10, 15, 21, 22]. Nonetheless, methenamine silver staining and immunofluorescence assay have lower sensitivity [15, 22]. Nested PCR and sequencing, compared with other staining methods, reportedly show the highest sensitivity and quality that is in agreement with the results of the present study [16, 17]. However, in a study performed on normal individuals, there was no difference between PCR and conventional methods in the diagnosis of* P. jirovecii* [23]. 

The *mtLUS* rRNA gene was selected for genotyping since it includes a high level of genetic conservation that is helpful for discovering intraspecific differences among populations [24]. *Pneumocystis* *jirovecii mtLUS* rRNA gene has a ‘C’ at site 248 and a ‘T’ at site 289; in addition, it has nucleotide variation just at site 85 [25]. Based on this base polymorphism, the isolates could be categorized into three distinct groups, namely genotype 1 which has a C, genotype 2 that has an A, and genotype 3 that has a T base at position 85 (Figure 3). 

Few studies have been conducted on other gene markers in Iran. The present study involved the investigation of *mtLUS* rRNA. Consistent with the results of a similar study performed in Shiraz by Badiee *et al*. [18], our results revealed genotypes III and II as dominant types. Badiee *et al*. reported genotypes III and II from tree positive cases. Additionally, the same result was reported in India by Gupta *et al. *[26]. In Italy, the genotyping of *P. jirovecii* in BAL samples obtained from patients in different clinical settings revealed genotypes III and II in Turin, and genotype II in Roma and Lebanon [27-29]. 

Furthermore, the same epidemiology study on patients with different pulmonary diseases in Spain showed a high prevalence of genotypes I and III [30]. In another study carried out in the United States and Cuba, most of the genotypes were distinguished as genotypes I and II [24, 31]. This polymorphism alteration suggests the regional variation of *P. jirovecii*.

Normal patients (without pneumocystis) and infected ones with immunodeficiency had similar clinical signs. However, the difference between sputum cough (productive) and dry cough was completely distinct between the two groups. In immunodeficient patients, *P. jirovecii* has been found in 10 out of 40 (25%) patients that is more than the rate observed in normal patients. Other studies reported that 2.5-20% of patients with medium to severe immunosuppression are infected by *P. jirovecii *[32, 33]. This finding is almost in agreement with the results of the current study.

In the present study, 43% of the patients were transplanted, 38% of the cases were corticosteroid users, and only one patient with HIV had *P. jirovecii *in his lung. Patients who receive immunosuppressive medications are more at the risk of infection with pneumocystis as corticosteroids change the cell surface layers, which in turn leads to easier infection dissemination [13]. 

In a study carried out by Maskell et al. [20] and Helweg-Larsen et al. [34], 44% and 75% of corticosteroid users were respectively reported to be affected by *P. jirovecii*, which is in agreement with our result (25%). Nevertheless, in a study by Khalife et al., corticoid therapy was not expressed as a risk factor [29]. 

In line with the results of the other studies [10, 23], in the current study,* P. jirovecii *was found in 7 (7.1%) patients out of 98 subjects with normal immune system, indicating that the general public could be a source and reservoir of infection [30]. People with a normal immune system can transmit the infection to others in a short period of time. Therefore, healthy people are the potential reservoir and source of human infection for all *P. jirovecii *species [20].

In our study, 6 out of 32 diabetic patients were infected by *P. jirovecii *(18.8%). High blood sugar decreases the defensive power of the body and opens the path for opportunist organisms. As a result, it is necessary to pay much more attention to this basic factor in diabetic patients. *Pneumocystis jirovecii *is an organism with very high clinical importance. This organism is more common in immunodeficient patients than in healthy people. Transplant patients and corticosteroid users are at the risk of PCP. Regarding this, patients with pulmonary diseases should be given special attention in terms of *P. jirovecii *infection. In our study, patients with a normal immune system could be a reservoir of *P. jirovecii*, thereby playing the role of a source of infection for patients with pulmonary diseases. 

## Conclusion

A direct experiment is not a suitable method for the diagnosis of *P. jirovecii *infection since the molecular methods, such as *mtLUS* rRNA sequencing, have much more sensitivity and specificity. In the current study, genotypes III and II were identified as the dominant genotypes. It is required to perform further studies to reveal the rout and genotyping of *P. jirovecii *based on *mtLUS* rRNA gene among the infected patients living in the different part of Iran, as well as in the Middle East. 
